# Metabolite-Sensing G Protein-Coupled Receptors Connect the Diet-Microbiota-Metabolites Axis to Inflammatory Bowel Disease

**DOI:** 10.3390/cells8050450

**Published:** 2019-05-14

**Authors:** Hassan Melhem, Berna Kaya, C. Korcan Ayata, Petr Hruz, Jan Hendrik Niess

**Affiliations:** 1 Department of Biomedicine, University of Basel, 4031 Basel, Switzerland; hassan.melhem@unibas.ch (H.M.); berna.kaya@unibas.ch (B.K.); korcan.ayata@unibas.ch (C.K.A.); 2 University Center for Gastrointestinal and Liver Diseases, St. Clara Hospital and University Hospital of Basel, CH-4031 Basel, Switzerland; petr.hruz@usb.ch

**Keywords:** inflammatory bowel diseases, metabolites, microbiota, metabolite-sensing G protein-coupled receptors

## Abstract

Increasing evidence has indicated that diet and metabolites, including bacteria- and host-derived metabolites, orchestrate host pathophysiology by regulating metabolism, immune system and inflammation. Indeed, autoimmune diseases such as inflammatory bowel disease (IBD) are associated with the modulation of host response to diets. One crucial mechanism by which the microbiota affects the host is signaling through G protein-coupled receptors (GPCRs) termed metabolite-sensing GPCRs. In the gut, both immune and nonimmune cells express GPCRs and their activation generally provide anti-inflammatory signals through regulation of both the immune system functions and the epithelial integrity. Members of GPCR family serve as a link between microbiota, immune system and intestinal epithelium by which all these components crucially participate to maintain the gut homeostasis. Conversely, impaired GPCR signaling is associated with IBD and other diseases, including hepatic steatosis, diabetes, cardiovascular disease, and asthma. In this review, we first outline the signaling, function, expression and the physiological role of several groups of metabolite-sensing GPCRs. We then discuss recent findings on their role in the regulation of the inflammation, their existing endogenous and synthetic ligands and innovative approaches to therapeutically target inflammatory bowel disease.

## 1. Introduction

Inflammatory bowel disease (IBD), which encompasses Crohn’s disease (CD) and ulcerative colitis (UC), is a chronic, relapsing inflammatory disorder of the gastrointestinal (GI) tract [1]. The inflammation affects the functioning of GI organs causing abdominal pain, persistent diarrhea, cramping, weight loss, gastrointestinal bleeding, and fatigue [2]. The prevalence of IBD has been continuously increasing during the last half-century and varies according to geographical location [3,4]. Like most autoimmune diseases, IBD has multifactorial causes, in which environmental factors, including, diet and gut microbiota, and the immune system, are gaining increased attention. In this regard, changes in the diet may contribute to the increased prevalence of IBD. Indeed, clinical pieces of evidence have reported that diets rich in poly-unsaturated fatty acids, trans-fats, alcohol, and red/processed meat may increase the risk of IBD [5,6]. By contrast, a diet rich in fibers reduces the CD risk [7]. 

Microbial products, such as polysaccharides and proteins can induce the intestinal immune system through the activation of pattern recognition receptors, including Toll-like receptors, and the nucleotide-binding oligomerization domain-like receptors (NLRs). Besides microbial products, other factors, including bacterial metabolites, can influence the immune response to commensals or pathogens. In this system, microbiota-derived metabolites serve as "signal molecules" and continuously contribute to the proper function of the gut by acting on the epithelium and immune cells. It has been estimated that 10% of metabolites, found in the mammalian blood stem from the gut microbiota [8], and these compounds, including short-chain fatty acids (SCFAs) primarily acetate, propionate and butyrate, indole and polyamines [9,10,11], are present in most host tissue [12]. 

The mechanisms that are influenced by dietary and bacterial metabolites are embedded in a complex signaling network. Host enzymes have the ability to directly digest some dietary products like the milk sugar lactose, which is digested into glucose and galactose by lactase. On the other hand, breakdown of certain molecules requires the involvement of microbiota. Dietary and host products are fermented by bacteria in the gut that produce metabolites, which in turn influences host immune responses. Moreover, an immune reaction against a pathogen will change the metabolism of the organism, as highlighted by a reduced immune response that is a evolutionarily conserved response to infections in warm- and cold-blooded organisms.

**Figure 1:**
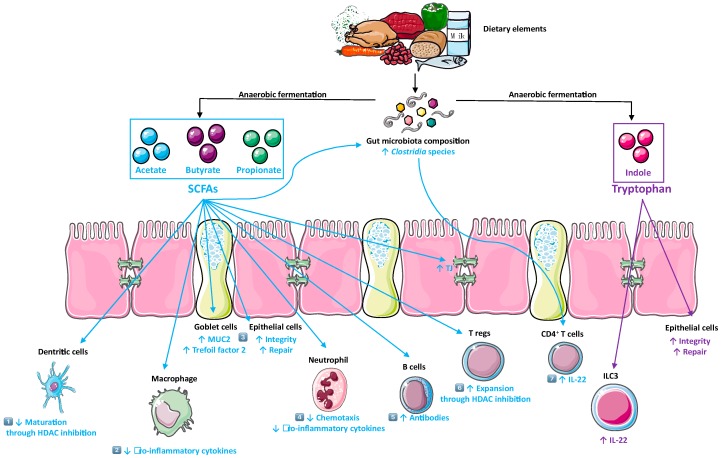
Dietary fibers are fermented by the gut microbiota that produce short chain fatty acids (SCFAs) (green). SCFAs exert anti-inflammatory effects, such as (1) inhibiting the maturation of dendritic cells, (2) reducing production of pro-inflammatory cytokines by innate immune cells, (3) promoting the intestinal barrier via epithelial cells and goblet cells, (4) reducing neutrophils infiltrate, (5) facilitating production of antibodies by B cells, (6) the expansion of regulatory T and (7) enhancing the epithelial integrity by increasing *Clostridia* species in the gut which promote IL-22 production by CD4^+^ T cells. Tryptophan is degraded to indole derivatives (purpule). Indoles exerts an anti-inflmmatory effects by promoting the intestinal epithelial barrier through supporting type 3 innate lymphoid cells, the major producers of IL-22. (adapted from SERVIER MEDICAL ART (CC of license 3.0)).

Microbial- or host-derived metabolites may act intracellularly either as histone deacetylase (HDAC) inhibitors, allowing the transcription of genes or as transcriptional coactivators (Figure 1). On the other hand, these metabolites may also act as extracellular signaling molecules through the metabolite sensing-G protein-coupled receptors (GPCRs) [9]. In this review, we focus on metabolite-sensing GPCRs and their ligands, which contribute to: (i) The regulation of intestinal immune system through inflammatory leukocytes, regulatory T (Tregs) cells, and (ii) the control of the intestinal barrier between gut microbes and host (action on nonimmune cells) [13]. Traditionally, GPCRs are receptors for hormones, neurotransmitters, and chemokines. However, this view is now changing as a growing number of metabolite-sensing GPCRs are being identified as receptors for metabolites, including fatty acid family members, which vary in carbon chain length, ranging from short chain fatty acids SCFAs (C2-5), medium-chain fatty acids (MCFAs) (C6-12), and long-chain fatty acids (LCFAs) (> C13); products of tryptophan metabolism and various others [9,10]. 

In this review, we first summarize the biology of the metabolite-sensing GPCRs. Second, we discuss recent findings demonstrating the impact of the receptor signaling and their metabolite ligands on IBD in order to explain, in part, the role of diet and metabolite-mediated inflammatory processes, and finally to provide new therapeutic strategies for the treatment or the prevention of IBD.

## 2. Diet as A Risk Factor for IBD: Epidemiological Studies 

The incidence of IBD is rising in industrialized countries [3,14]. Westernization of lifestyle is associated with changes in diet, hygiene status, antibiotic use, microbial exposure, and pollution. These factors have been associated with the development of IBD [15]. However, diet is one of the potentiel environmental factors that may link industrialization and the western lifestyle to the increased incidence of IBD [16]. Numerous large prospective cohort studies have attempted to identify dietary patterns that contribute to the risk for IBD. The Nurses’ Health Study (NHS) showed that people who consume a high amount of fiber, mainly fruits, are less susceptible to developign CD [7]. Findings from these cohorts showed an inverse correlation between the risk of CD and the intake of potassium and zinc [17,18].

Furthermore, the NHS showed that higher consumption of high omega-3 (n-3) to omega-6 (n-6) polyunsaturated fatty acid (PUFA) ratio is protective against the development of UC [5]. Similarly, the European Investigation into Cancer and Nutrition Study showed that individuals who consume high amounts of red meat, which contains a high concentration of linoleic acid (an n-6 PUFA), have a higher incidence of UC [19].

## 3. Diet as A Modulator of Gut Microbiota and Their Metabolites 

Increasing evidence suggests that the diet influences the composition of the gut microbiome and the metabolites produced by the microflora. Indeed, breastfed infants develop a different gut microbiota, at initial colonization, compared to infants fed with a formula diet [20]. The inhibition of the immune response, during colonization, may predispose people to colitis susceptibility, allergy, and cancer later in life [21]. In adults, dietary patterns have been proposed to alter the composition of the intestinal microbiome [22]. The major variation of gut microbiota is related to dietary changes, indicating the dominant role of diet in shaping bacterial composition [23,24]. In IBD patients, the occurrence of dysbiosis has been observed. A high-fat and low-fiber diet can leed to dysbiosis in healthy volunteers [25,26], indicating that the diet is a major determinant of the microbiota. A vegetarian diet rich in fibers prevents the growth of potentially pathogenic bacteria, such as *E. coli,* in human-mediated by the production of SCFA, which decreases the intestinal pH [27]. In mice, a dietary haem iron, which is associated with changes in the gut microbiota, [28] induces oxidative stress-mediated colonic epithelium injuries [29]. Moreover, the switch from a low-fat, plant polysaccharide-rich diet to a high-fat, high-sugar "Western" diet, may causes a dysbiosis [30], indicating that there are multiple forms of diets that can influence the composition of the microbiota, with a potential impact on the development of intestinal diseases. Thus, a deeper understanding of receptors that recognize microbial-derived metabolites may help to identify factors leading to IBD.

## 4. GPCRs Sense Microbial-Derived Metabolites

Metabolite-sensing GPCRs can bind to metabolites derived from consumed foods. These metabolites are produced either, by direct host metabolism, or digestion, such as MCFAs (derived from coconut, palm kernel, and milk), LCFAs (derived from olive oil and fish), niacin and kynurenic acid (intermediates of tryptophan metabolism by the host) or by secondary metabolites after gut bacterial fermentation. These include, for example, SCFAs (derived from fermentation of fiber diet by gut flora) and indole-3-aldehyde (derived from bacterial metabolism of tryptophan). 

SCFAs are generated mostly in the colon from undigested carbohydrates, including plant polysaccharides and soluble oligosaccharides after fermentation by the anaerobic microbiota community [31,32]. The major products are acetate (C-2), propionate (C-3), and butyrate (C-4) [33,34]. Protein fermentations may also contribute to SCFAs production [35]. However, carbohydrates are the most significant sources. The synthesis of the three SCFAs acetate (C-2), propionate (C-3), and butyrate (C-4) depends on the composition of the microbiota and environmental conditions, such as pH, available substrates, and hydrogen partial pressure [36,37]. SCFAs are transported by monocarboxylate transporter 1 (MCT1) and sodium-coupled monocarboxylate transporter 1 (SMCT1) across the cell membrane of epithelial cells into the host. Butyrate can be used as a significant energy source by colonocytes and is primarily metabolized within the epithelial mucosa. Acetate travels intact to the liver and then it is released into systemic circulation [38]. Propionate exhibits an average distribution [11]. MCFAs are used mainly by the liver as a source of energy after absorption through the duodenum. Enterocytes take-up LCFAs and esterify LCFAs into complex lipids to serve as an integral part of the cell membrane, as a metabolic fuel, as precursors of lipid mediators, as regulators of ion-channels, and as modulators of gene expression [39].

Moreover, tryptophan metabolites derived from the host and microbiota metabolism bind to metabolite-sensing GPCRs. Tryptophan is an essential amino acid required for protein biosynthesis. Also, tryptophan functions as a precursor for serotonin, an important neurotransmitter synthesized in the gut and in the brain. The liver regulates tryptophan metabolism by the degradation of excess tryptophan. In the intestine, tryptophan metabolites such as kynurenine, indole-3-aldehyde, and indole-3-acetic are known to regulate inflammation by acting on the adaptive immunity and the intestinal barrier. 

## 5. Metabolites and Mucosal Inflammation 

There is now overwhelming evidence about positive benefits of bacterial metabolites particularly in the gut (Figure 1) where they are present at the concentration of 60 mM, while in peripheral organs the concentration of these metabolites are significantly lower (80 μM and 3 μM for acetate, and butyrate, respectively) [40].

### 5.1. SCFAs 

In IBD patients, lower levels of SCFAs have been reported, compared to healthy subjects [41,42]. Furthermore, reduced numbers of SCFAs-producing bacteria (Bacteroidetes and Firmicutes) were observed in IBD patients [43]. Moreover, the two SCFAs transporters (MCT1 and SMCT1) are down-regulated during inflammation and in the inflamed mucosa of IBD patients [44,45]. On the other hand, the administration of SCFAs reduces the human colitis severity [46,47,48]. Friedrich and co-workers [49] recently showed that Givinostat, a potent HDAC inhibitor, improved barrier recovery and epithelial wound healing in vivo and in vitro. With regards, SCFAs, such as butyrate, exert its anti-inflammatory effects via the inhibition of HDAC, leading to hyperacetylation of histones with suppression of nuclear factor kappa B on activated lamina propria macrophages isolated from patients with ulcerative colitis [50]. In animal models, low-fiber diets aggravate DSS-induced colitis while high-fiber protects from colitis [51]. Consistent with these findings, multiple in vivo and in vitro studies indicate that intracellular SCFAs, mainly butyrate may have beneficial effects on gut health. Indeed, butyrate reinforces the intestinal epithelial barrier by stimulating the production of MUC2 as well as tight junction (TJ) proteins [52,53,54]. Furthermore, several studies showed that butyrate decreases the severity of colitis by increasing the expression of trefoil factor 3, a protein synthesized by IECs that initiate and improve wound healing after mucosal injury [55,56]. Consistently, butyrate manifests its anti-inflammatory properties by influencing intestinal immune cells, such as dendritic cells [57], macrophages, and T cells [58]. In this regard, butyrate leads to the downregulation of pro-inflammatory cytokines produced by LPS-stimulated macrophages that polarize T cells such as IL-23 and IL-12p70 and IL-6 [59,60]. Furthermore, butyrate and propionate seem to play a role in inhibiting the maturation of dendritic cells (DCs) by inhibition of HDAC in DCs [61,62]. Butyrate and propionate promote B cell differentiation into antibody-producing cells by accelerating metabolism and regulating gene expression [63]. Butyrate also modulates mucosal inflammation by regulating colonic Tregs [64]. The inhibition of HDACs by butyrate induces the differentiation of Tregs, by increasing the acetylation of histone H3 of FOXP3 promoter [65,66,67,68,69]. A more recent study [70] reported that butyrate acts via its HDAC3 inhibitory function to alter metabolism and induce production of anti-microbial peptides, leading to enhanced bactericidal function in vitro and in vivo. Another mechanism by which butyrate reduces intestinal inflammation is through the reduction of cell infiltration, by decreasing the expression of surface receptors for chemoattractants such as C5aR and CXCR2 [71]. 

### 5.2. Tryptophan Metabolites 

In a murine model of colitis, dietary supplementation with tryptophan and tryptophan metabolite reduces the signs of colitis [72]. Conversely, mice fed with a tryptophan-deficient diet are more susceptible to the develop colitis [72]. Moreover, IBD patients exhibited lower levels of serum tryptophan, as compared to healthy controls [73]. Additionally, mRNA expression level of Indoleamine 2,3 dioxygenase-1, the first step in the kynurenine pathway, is upregulated in human and murine colonic tissues [73,74]. Microbes use tryptophan as an energy source to produce ligands for the aryl hydrocarbon receptor (AhR) [75], which is necessary for the maintenance of the epithelial barrier and the proper function intraepithelial lymphocytes (IELs) [76,77]. Indeed, loss of AhR aggravates the intestinal inflammation in mice treated with DSS and the transfer of IELs to AhR^−/−^ mice reduces the signs of colitis [77]. Furthermore, AhR^−/−^ mice are susceptible to infections with the bacteria *Listeria monocytogenes*, *Citrobacter rodentium* and with the fungus *Candida albicans* [76,78,79]. Protection against these pathogens is mediated by IL-22, which requires the AhR signaling [77,80,81]. 

## 6. Metabolite-Sensing G Protein-Coupled Receptors

Outside of the cells, metabolites can function as agonists for several metabolite-sensing GPCRs (Table 1). These receptors constitute the most abundant protein family of membrane proteins in mammals. Recently, multiple studies pointed out the importance of these receptors in maintaining physiological functions especially in the gut. When a ligand binds to metabolite-sensing GPCRs, it causes a conformational change. Thus, the receptors can activate an associated G protein. The G protein α subunit, can then dissociate from the β and γ subunits to further trigger intracellular signaling proteins or target functional proteins directly depending on the α subunit type (G_αs_, G_αi/o_, G_αq/11,_ and G_α12/13_) and β-arrestins, including cyclic-adenosine monophosphate (cAMP), phospholipase c-β, and RhoGEFs.

Moreover, these receptors regulate downstream signaling pathways including, different kinase cascades, such as ERK/MAPK, JNK, p38, or the Akt/PI3K to control cell proliferation, differentiation, survival, and migration [82,83,84]. We will further discuss the respective GPCRs identified over the past years and their signaling pathways in the context of IBD below. 

### 6.1. GPCRs for Short Chain Fatty Acids (SCFAs)

**GPR41.** GPR41 is mainly activated by propionate, acetate, caproate, butyrate, and valerate to a lesser degree [85]. Activation of this receptor leads to intracellular Ca^2+^ release and reduction of cAMP [86]. Studies reported that GPR41 is expressed in the colonic epithelium [85,87], and also in spleen and the pancreas at low levels. In adipocytes, the expression of GPR41 remains controversial. In animals, activation of GPR41 by propionate leads to altered bone marrow hematopoiesis with affected Th2 cell development in models of allergic disease [88]. GPR41 also regulates blood pressure through renin release. In murine intestinal epithelial cells, the activation of GPR41 induced the mitogen-activated protein kinase signaling and production of chemokines and cytokines. These pathways mediate protective immunity and tissue inflammation [89].

**GPR43.** Acetate and propionate are fermentation products of anaerobic bacteria in the gut, and both have equal affinity for GPR43. In vitro activation of GPR43, induces the release of Ca^2+^ intracellularly and reduces cAMP in the colon epithelium [85,90]. Human neutrophils, macrophages, dendritic cells, intestinal epithelial cells express GPR43 [90,91,92,93]. Signaling via GPR43 downregulates inflammatory responses. Genetic ablation of GPR43 in mice promotes allergic airway disease. Further analysis showed that GPR43 also suppresses insulin-mediated fat accumulation in adipose tissues [94]. Additionally, the absence of GPR43 impaires β cell proliferation leading to loss of glucose tolerance and impaired insulin secretion [95]. Recently, Kim and co-workers demonstrated that deficient GPR43 mice were defective in mounting appropriate immune responses to promote barrier immunity, and develop uncontrolled chronic inflammations following epithelial damage [96].

**GPR109A and GPR109B.** Although niacin was identified as the first GPR109A agonist, later studies demonstrated that the SCFA butyrate might be more biologically relevant for this receptor, in the gut, where the level of butyrate is sufficiently high. However, in the periphery, butyrate is present at a lower level than in the gut, which would question the relevance of this agonist. GPR109A is detectable on macrophages, neutrophils, and adipocytes. Additionally, in mice, GPR109A is expressed in the small and large intestine and broadly in the spleen and bone marrow. Activation of GPR109A by niacin reduces the secretion of pro-inflammatory cytokines by macrophages, monocytes, and epithelial cells [97,98,99]. In mice that lack GPR190A, the nicotinic acid-induced decrease in free fatty acid and triglyceride plasma levels was abrogated, indicating that GPR190A mediates the anti-lipolytic and lipid-lowering effects of nicotinic acid in vivo [100]. Moreover, GPR109A knock-out mice are characterized by an inflammation in the DSS-induced colitis model [51,57].

Although GPR109B and GPR109A share high homology, GPR109B and GPR109A do not share the same ligand niacin. D-tryptophan and the essential amino acid D-phenylalanine can bind to GPR109B [101]. Like GPR109A, GPR109B appears to play a role in the immune system. Indeed, aromatic D-amino acids elicit a chemotactic response in human neutrophils via activation of GPR109B [101]. Additionally, data shows that the 3-OH-octanoic acid and GPR109B mediate a negative feedback regulation of adipocyte lipolysis [102]. 

### 6.2. GPCRs for Medium and Long Chain Fatty Acid (MCFA, LCFA)

**GPR40.** Saturated and unsaturated fatty acids with a chain length of C8–C22 [103] activate GPR40, which is predominately expressed in the pancreas and the liver [103]. Moreover immune cells, taste buds, and the central nervous system also express GPR40 [104,105,106]. Fatty acid binding to GPR40 appears to activate the G_αq/11_-protein complex, resulting in the activation of phospholipase C. Activation of GPR40 plays an essential role in the regulation of insulin secretion. However, experiments on the function of this receptor have produced conflicting data. In vitro, the reduction of GPR40 expression by siRNA in β cell lines or isolated mouse islets reduced fatty acid augmentation of insulin secretion [107,108]. Comparable results have been observed in GPR40^−/−^ mice [109,110]. Conversely, overexpression of GPR40 in pancreatic β cells augmented glucose-stimulated insulin secretion and improved glucose tolerance in normal and diabetic mice [111]. In the mouse gut, 10-hydroxy-cis-12-octadecenoic acid, a bacterial metabolite of linoleic acid, ameliorated the intestinal epithelial barrier impairment partially via GPR40-MEK-ERK pathway [112].

**GPR120.** GPR120, which shares 10% amino acid homology with GPR40, is identified as a receptor of omega-3 fatty acids, but in vitro, omega-6 fatty acids can also bind this receptor [113]. Hirasawa et al. showed that the activation of GPR120 by FFAs resulted in the elevation of intracellular Ca^2+^ and activation of the ERK cascade, which suggests an interactions with the Gαq family of G proteins [114]. The same study showed that this receptor is highly expressed in the intestinal epithelium particularly in the enteroendocrine [114]. Additionally, GPR120 was detectable in macrophages, adipocytes and type II taste cells [105,115,116]. Ichimura and colleagues have reported that GPR120-deficient mice fed with a high-fat diet develop obesity, glucose intolerance and fatty liver with decreased adipocyte differentiation and lipogenesis and enhanced hepatic lipogenesis [117]. 

**GPR84.** MCFAs, with a chain length of 9-14 carbons, exclusively bind to this receptor [118]. The GPR84 receptor is expressed by adipose tissue and by various leucocyte types, particularly macrophages and neutrophils and its expression is increased under inflammatory conditions [118,119]. Although the reported evidence showed that the absence of GPR84 selectively affects IL-4 production in CD4^+^ T cells [120], the biological relevance of this receptor remains poorly understood.

### 6.3. GPCRs for Amino Acids and Related Metabolites

**GPR35.** GPR35 is a poorly characterized GPCR expressed in the GI tract particularly in the stomach, intestinal epithelial cells, DCs, and macrophages of the small intestine and colon [121]. Endogenous ligand candidates for GPR35 include kynurenic acid and 2-oleoyl lysophosphatidic acid while pamoic acid, zaprinast and YE120 are synthetic GPR35 agonists [122,123,124,125,126]. Furthermore, CXCL17, a chemokine that is crucial for homing of alveolar macrophages, was also proposed as a ligand for GPR35. However, another study showed that CXCL17 neither activates GPR35 signaling nor induces chemotaxis in GPR35-expressing cells [127,128,129]. Therefore, CXCL17 remains controversial in this context. The identification of the T108M missense single nucleotide polymorphism by genome wide association studies (GWAS) as a risk factor associated with UC and CD [130,131] suggest that GPR35 play an essential role in intestinal homeostasis. 

### 6.4. Bile Acid-Sensing GPCRs

**GPR131.** GPR131 also named TGR5 was discovered in 2002 [132] and it is highly expressed in immune cells (monocytes and macrophages), muscle, spinal cord, adipocytes, and the enteric nervous system [133]. This receptor is particularly relevant for gut macrophages and monocytes because it can recognize primary and secondary bile acid metabolites [134]. Lithocholic acid and taurolithocholic acid, as secondary bile acids, are the most potent ligands of TGR5 [135]. In murine macrophages specific deletion of TGR5 leads to insulin resistance [136]. Moreover, genetic ablation of TGR5 aggravated the intestinal inflammation in DSS- and TNBS-induced colitis [137]. Guo et al. showed that TGR5 exerts an anti-inflammatory effect through NLRP3 inflammasome activation [138].

### 6.5. pH-Sensitive Receptor

**GPR65.** Protons in extracellular acidic conditions bind to GPR65 [139] and act as surrogate receptors for acidic metabolites, such as SCFAs [13]; however, no evidence has verified this hypothesis. GPR65 is expressed on a variety of immune cells, including T and B lymphocytes, neutrophils, but is unusually high on eosinophils and mast cells. GWAS studies have associated single-nucleotide polymorphisms in GPR65 to increased IBD susceptibility [140,141]. Multiple pieces of evidence have shown the cytoprotective effect of GPR65, since increased viability of eosinophils has been associated with activation of GPR65 under acidic conditions [142,143]. Furthermore, the expression BCL2 (antiapoptotic protein B cell lymphoma 2) correlates with the expression of GPR65 in chronic lymphatic leukemia cells [144]. 

### 6.6. GPCRs for Citric Acid Cycle Intermediates

**GPR91.** The GPR91 (SUCNR1) receptor is activated by succinate coupled to G_i_-type G proteins [145]. The liver, heart, adipose tissue, intestine, spleen, and immune cells, including DCs express GPR91 [108,146,147,148]. GPR91 has a possible role in renovascular hypertension. Indeed, the succinate-induced hypertensive effect involves the renin-angiotensin system and is abolished in GPR91-deficient mice [145]. In the immune system, GPR91 appears to play a pro-inflammatory role. In this regard, DCs expressing GPR91 enhance their immuno-stimulatory potential in a GPR91-dependent manner [147]. Furthermore, GPR91-deficient mice have a reduced production of IL-1β by macrophages during antigen-induced arthritis [149]. 

**GPR31.** GPR31 expressed in macrophages (133) has been identified as a target receptor for 12(S)-hydroxyeicosatetraenoic acid [150]. GPR31 plays a crucial role in inflammation and tumor progression [151]. Possibly, lactate and pyruvate, contribute to enhanced immune responses by inducing GPR31-mediated dendrite protrusion of intestinal CX3CR1+ phagocytes [152]. Moreover, elevated GPR31 expression levels were reported in colorectal cancer tissue as compared to normal mucosa [153]. 

## 7. Metabolite-Sensing GPCRs and Inflammatory Bowel Disease

The use of axenic mice is one of the most straightforward examples that shows the importance of commensal organisms in the development of a functional immune system [154,155]. However, the exact molecular mechanisms by which the gut flora shapes the immune system remain to be explored. On the other hand, increasing evidence showed that bacterial-derived metabolites play a crucial role in the development of the immune system via metabolite-sensing GPCRs. 

Metabolite-sensing GPCRs are present in immune, mesenchymal, and epithelial cells of the gut, where their ligands are synthesized in high concentrations by the gut microbiota or derived from nutritional content. GWAS studies have established a link between IBD and the metabolites sensing-GPCRs, in this context, GPR35 [121] and GPR65 [140,141] are considered among the IBD-risk genes. Clinically, administration of SCFAs, mainly butyrate ameliorated intestinal inflammation [46,47,156] is by signaling through metabolite-sensing GPCRs. Macia et. al [51] have shown that dietary fiber protects from DSS-induced colitis via GPR43 and GPR109A in mice. Furthermore, loss of GPR35 in knockout mice aggravated the intestinal inflammation in DSS-induced experimental colitis [157]. Additionally, the activation of GPR35 by pamoic acid significantly reduced the severity of DSS-induced colitis [158]. Another study has reported that GPR41^−/−^ and GPR43^−/−^ mice had reduced inflammatory responses after administration of ethanol or TNBS compared to control mice, and had a limited immune response against *C. rodentium* infection [89]. Similarly, the loss of GPR43 in mice exacerbated inflammation in DSS-induced colitis and the administration of acetate reduced the severity of colitis in wild-type mice but not in mice lacking GPR43, suggesting that acetate exerts its anti-inflammatory role via GPR43 [71]. Furthermore, epithelial cells expressing the IBD-associated missense variant, GPR65 I231L, displayed aberrant lysosomal pH, resulting in lysosomal dysfunction and impaired bacterial restriction [159].

Despite the high number and quality of pieces of evidence supporting the anti-inflammatory functions of metabolite-sensing GPCRs, other studies showed that some metabolite-sensing GPCRs might exert a pro-inflammatory role in the gut. The production of IL-8 and TNF-α by LPS-stimulated neutrophils and macrophages respectively was increased by activation of GPR84 with natural and synthetic agonists [160]. Similarly, GPR91 plays a pro-inflammatory role as it has been demonstrated that LPS stimulation of macrophages increases the levels of succinate, which subsequently drives IL-1β production by macrophages [161]. 

## 8. Possible Mechanisms whereby Metabolite-Sensing GPCRs Exerts an Anti-Inflammatory Effect in the Gut 

**Figure 2:**
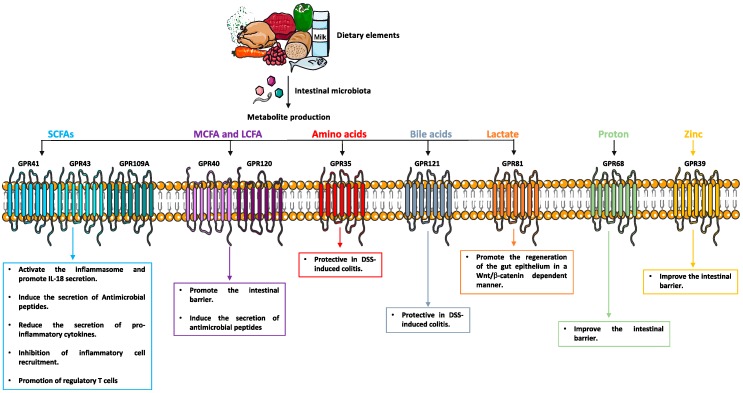
Metabolites are produced by the microbiota and also host cells in the gut. Metabolite-sensing G protein-coupled receptors (GPCRs) are highly expressed in intestinal immune and nonimmune cells that recognized microbiota-derived metabolities Activation of these metabolite-sensing GPCRs promotes intestinal homeostasis through enhancing the intestinal epithelial barrier (activation of inflammasome, increasing the expression level of tight junction proteins, promotion of intestinal stem cells and secretion of antimicrobial peptides), decreasing the production of the inflammatory cytokines, reducing the cell infiltrates and activation of regulatory T cells (adapted from SERVIER MEDICAL ART (CC of license 3.0)).

A general model on how metabolite-sensing GPCRs may impact IBD is shown in Figure 2. The primary two anti-inflammatory mechanisms by which metabolite-sensing GPCRs maintain intestinal homeostasis include: (1) regulation of the gut epithelium and (2) promotion of both innate and adaptive intestinal immunity. 

### 8.1. Metabolite-Sensing GPCRs Enhance Epithelial Integrity

**Inflammasome.** The inflammasome and the secretion of IL-18 are some of the critical mechanisms by which intestinal epithelial homeostasis is maintained [162,163,164]. In this regard, mice lacking NLRP3 exacerbated colitis in the DSS model [162,163]. However, the role of NLRP3 inflammasome in colitis is controversial. Indeed, hyper-activation of the NLRP3 inflammasome can lead to colitis in different contexts [165]. In a mouse model of colitis, SCFAs decreased the severity of colitis by activating NLRP3 as well as IL-18 secretion through GPR43 and GPR109A [51,57]. The uses of bone marrow chimera model by Macia et. al [51] showed that the beneficial effect of GPR43 is related to its expression in epithelial cells. It has been reported that Ca2^+^ [166,167] mobilization or K^+^ efflux can activate the inflammasome [168]. In this regard, induction of Ca2^+^ mobilization or K^+^ efflux upon activation of GPR43 or GPR109A may initiate activation of the inflammasome. However, this observation requires further investigations. 

**Intestinal barrier.** GWAS and animal studies have shown that the dysfunction of TJ in the intestine contributes to the pathogenesis of IBD [169,170] as the disruption of zonula occludens-1 (ZO-1) and occludin leads to an increase in intestinal permeability and a decrease in transepithelial resistance [171]. Multiple studies have evaluated the role of metabolite-sensing GPCRs in regulating the intestinal barrier. Indeed, the microbial metabolite linoleic acid (10-Hydroxy-cis-12-octadecenoic acid), ameliorates intestinal epithelial barrier impairment via GPR40-MEK-ERK pathway [112]. Recently, Shao et al. showed that the activation of GPR39 by zinc improves the epithelial integrity by enhancing the abundance of ZO-1 in *S. typhimurium*-infected Caco-2 cells [172]. Furthermore, the activation of GPR68 by an acidic extracellular environment improved barrier function, and inhibited cell migration and proliferation, during the in vitro model of wound healing in Caco-2 cells [173].

**Intestinal stem cells.** The differentiation of the intestinal stem cells into functional intestinal epithelium cells, including Paneth cells, goblet cells, enteroendocrine cells, tuff cells and enterocytes which allows the self-renewal of the intestinal epithelium [174]. On the other hand, the Wnt pathway plays an indispensable role in supporting the stem cell self-renewal [175]. Recently, the role of bacterial metabolites in the promotion of the intestinal stem cell niche has been studied. Lee and co-workers [176] showed that activation of GPR81 expressed on Paneth and stromal cells by symbiont-derived lactate promotes the regeneration of the gut epithelium in a Wnt/β-catenin dependent manner. 

**Antimicrobial peptides.** Antimicrobial peptides (AMPs) are critical components of innate immunity with antimicrobial activities and are pivotal for intestinal defense. AMPs are expressed either in a constitutive or in an inducible manner in response to microbial invasion [177]. Zhao et al. [178], found that expression of RegIIIγ and β-defensins 1, 3, and 4 is lower in intestinal epithelial cells in GPR43^−/−^mice compared to WT mice. Moreover, SCFA induces RegIIIγ and β-defensins in intestinal epithelial enteroids generated from WT but not GPR43^−/−^mice confirming that SCFA-induces antimicrobial peptides mediated through GPR43. 

### 8.2. Metabolite-Sensing GPCRs Promote the Intestinal Immune System

**Pro-inflammatory cytokines.** Part of the beneficial effects of metabolite-sensing GPCRs is related to the inhibition of pro-inflammatory cytokines secretion. GPR43-deficient mice are significantly more susceptible to colitis induced by DSS [71] which is related to increased production of inflammatory cytokines by *GPR43^−/−^* immune cells [71,179].

Furthermore, activation of GPR109A by nicotinic acid in LPS-stimulated human monocytes, reduces the production of pro-inflammatory cytokines including TNF-α and IL-6 [97]. Similarly, stimulation of GPR120 with omega-3 fatty acids decreases the secretion of pro-inflammatory cytokines by monocytic and primary intraperitoneal macrophages, moreover these effects were reversed in GPR120 deficient mice [115].

**Inflammatory cell recruitment.** Metabolite-sensing GPCRs manifest their anti-inflammatory effect through inhibition of inflammatory cell recruitment. Genetic ablation of GPR43 exacerbates the neutrophil migration and the treatment with acetate reduces neutrophil migration in WT mice, but not in GPR43^−/−^ animals [180]. 

**Regulatory T cells.** The essential anti-inflammatory role of metabolite-sensing GPCRs is perhaps their promotion of Tregs, which leads to the suppression of the activity of effector T cells. Several studies asked whether bacterial metabolites may regulate Tregs in the gut through metabolite-sensing GPCRs. Dietary fibers can restore colonic Tregs numbers in germfree mice and increase their numbers in colonized gnotobiotic mice [64] mediated through GPR43 expressed on colonic Tregs. Consequently, the feeding of mice with SCFAs protects against experimentally induced colitis in a GPR43 dependent manner [64]. GPR109A also appears to play a role in maintenance of Tregs. Indeed, it has been showed by Singh et al. [57] that colonic lamina propria of GPR109A^−/−^ mice harbor significantly less (~40%) frequency and count of Tregs cells compared to WT. Mechanistically, the Singh et al. study demonstrated that colonic DCs, master regulator of Treg induction, from GPR109A^−/−^ mice were defective in inducing differentiation of naïve OT II CD4^+^ T cells into Tregs cells [57].

## 9. Potential Tools for Studying Diet and Bacterial-Derived Metabolites in the Context of IBD

A diverse gut microbiota facilitates the establishment of a more stable host system, but this diversity leads to a complex system, which is a disadvantage for scientists who aim to investigate specific metabolites associated with defined species. The colonization of mice with defined consortia of microorganisms modulates the immune system of the host, which also influences the establishment and propagation of the microbiota. Some of the most powerful tools for research are the axenic and gnotobiotic mice, allowing for controlled in vivo investigation of the interaction between a specific strain and the host, by eliminating background signals coming from the rest of the gut microbiome. The effect of a specific diet in gnotobiotic animals colonized with a defined single or a consortium of microorganism can be investigated in this well-defined experimental setup. On the other hand, it is esential to keep in mind that gnotobiotic mice model and other factors (including birth mode of delivery, feeding’s mode (breast or bottle), diet, medical history, and social activities, which shape the human microbiota differently than the murine microbiota, does not reflect a ‘real-life’ gut microbiota. Furthermore, germ-free and gnotobiotic mice have an altered structure of the intestine as highlighted by an enlarged ceocum. Genetically modified animals that harbor inducible deletions of metabolite-sensing GPCRs known to modulate microbiota-host interactions, could serve as an alternative to study underlying mechanisms. The application of single-cell RNA sequencing significantly increased our perception to grasp relevant pathways associated with metabolite signaling. The next step would be to apply in vitro approaches by using cell lines and patient-derived organoids to monitor the effect of specific metabolites. Implenting single or multiple deletions of candidate GPCRs, and associated signaling partners, by using siRNA or Crispr/Cas9 methods in combination with varying doses of metabolites, will help in the understanding of mechanistical functioning of both ligands/receptors and signaling pathways.

## 10. Metabolite-Sensing GPCRs for the Treatment of IBD: What Are the Challenges?

The opportunity to manipulate the host immune system, through metabolite-sensing GPCRs, by small-molecules, will offer excellent potential for the treatment of IBD. However, metabolite-sensing GPCRs research comes with many challenges. In this regard, bacterial metabolites, such as butyrate, can exert anti-inflammatory effects either through HDAC inhibition or through metabolite-sensing GPCRs. Furthermore, metabolite-sensing GPCRs also signal through β-arrestin-2, which typically produces anti-inflammatory effects, and not only through G-proteins. Thus, it is essential to understand whether bacterial metabolites exert beneficial effects through HDAC inhibition, β-arrestin-2 signaling or signaling through G-proteins. In order to define possible treatments for IBD each of these different pathways needs to be further studied. 

The metabolite-sensing GPCRs GPR41, GPR43, and GPR109A can recognize with high affinity different bacterial metabolites (e.g., butyrate) and the same ligand can bind to different GPCRs indicating redundance in this system. Moreover, SCFAs, can alter local pH and possibly activate pH-sensing GPCRs as well along with their main GPCRs. Further studies are needed to dissect this highly redundant system before target therapies can be developed. 

The vast and varied systemic expression pattern and function of some metabolite-sensing GPCRs is another challenging aspect. Indeed, activation of GPR109A by niacin induces flushing in dermal Langerhans cells through increases of prostaglandin D2 and prostaglandin E2 and causing cutaneous vasodilatation [181]. Moreover, many GPCRs ligand can activate different pathways. For example, β-2-adrenergic receptor, agonists for the arrestin/MAP kinase pathway are also inverse agonists for the classical Gαs/cAMP/PKA pathway. Despite these confusing aspects, pharmacological compounds and existing ligands for targeting specific GPCRs provide a useful toolbox together with genetic models to solve these issues.

## 11. Conclusions

Shortly after their discovery, metabolite-sensing GCPRs became a favorable target for the development of new therapeutic tools. Most of these metabolite-sensing receptors are expressed in the gut where metabolites are produced in large numbers and amounts. Accordingly, recent high-profile publications pointed out their importance in regulating intestinal inflammation. A deeper understanding of how diet and metabolites, including bacteria- and host-derived metabolite and their GPCRs influence the intestinal homeostasis, and will undoubtedly be important in the elucidation of connections between diet, microbiome, and IBD, allowing the development of new approaches for the prevention and the treatment of patients with IBD.

## Figures and Tables

**Table 1:** Summary of metabolite-sensing G protein-coupled receptors (GPCRs).

Receptor	Ligands	G Protein	Main Expression	Effects on Immune System	Effects on Metabolism	Key IBD-Related Findings
**Short chain fat acid**						
GPR41 (FFAR3)	SCFAs (C2–C6): butyrate, acetate, propionate, valerate	Gαi	Immune cells: peripheral blood monocular cells and macrophages. Enteroendocrine cells, adipocytes, pancreatic islets	DC cells maturation, Th2 development, anti-inflammatory	Regulation of energy balance	Protective effect in DSS and TNBS-induced colitis
GPR43 (FFAR2)	SCFAs (C2–C6): butyrate, acetate, propionate, valerate, formate,	Gαi/Gαq	Immune cells: neutrophils, eosinophils. Enteroendocrine cells adipocytes, pancreatic islets	Tumor suppressor, anti-inflammatory	Loss of glucose tolerance, Impaired insulin secretion	Protective effect in DSS and TNBS-induced colitis
GPR109A (NACR1)	SCFAs (C4–C8): butyrate and nicotinic acid (niacin)	Gαi/Gαq	Immune cells: DC, neutrophils and macrophages. Intestinal epithelial cells, adipocytes	Inhibition of pro-inflammatory cytokines secretion, anti-inflammatory	Anti-lipolytic	Protective effect in DSS-induced colitis
**Medium and long-chain fat acid**						
GPR40 (FFAR1)	C12–C18: medium and long-chain fatty acids	Gαq/11	Pancreatic cells Enteroendocrine K cells	Anti-inflammatory	Regulation of insulin secretion and glucose tolerance	Protective effect in DSS-induced colitis, Ameliorate intestinal epithelial barrier
GPR84	C9–C14: medium-chain fatty acids	Gαi	Immune cells: leucocytes, neutrophils and macrophages. Adipocytes	Unclear	Unclear	Unstudied
GPR120 (FFAR4)	(C12–C22): long-chain fatty acids, unsaturated, ω-3 and ω-6 fatty acids	Gαi/Gαq	Immune cells: macrophages, DC, eosinophils Adipocytes and colon enteroendocrine cells	Inhibition of pro-inflammatory cytokines secretion	Regulation of insulin secretion	Protective effect in IL-10^−/−^ induced chronic colitis, Induce the secretion of antimicrobial peptides
**Amino acids and related metabolites**						
GPR35	kynurenic acid, lysophosphatidic acid and pamoic acid	Gαi	Immune cells: DC, monocytes, neutrophils, macrophages. Intestinal epithelial cells and nervous tissues	Leukocyte recruitment	Unstudied	SNPs associated with IBD, Protective effect in DSS-induced colitis
**pH-sensitive receptors**						
GPR65 (TDAG8)	Protons (H^+^)	Gαs	Immune cells: blood leucocytes. Spleen, thymus, lung and gut	Increase eosinophil viability	Unstudied	SNPs associated with IBD, Protective effect in DSS-induced acute and chronic colitis
**Succinate**						
GPR91 (SUCNR1)	Succinate	Gαi/Gαq	Immune cells: DC, macrophages and Platelets. Adipocytes, retinal neurons, liver, heart, intestine and spleen	Migration of Langerhans cells, hematopoiesis,	Hypertensive effects, activation of renin-angiotensin system	Unstudied
**Bile acid receptors**						
GPR131 (TGR5)	Lithocholic acid and taurolithocholic acid	Gαs	Immune cells: monocytes and macrophages. Muscle, adipocytes and enteric nervous system	Inflammasome activation, inhibit production of pro-inflammatory cytokine	Insulin resistance	Protective effect in DSS and TNBS-induced colitis.

## Abbreviations

IBD: Inflammatory bowel disease; CD, Crohn’s disease; UC, ulcerative colitis; GI, gastrointestinal; NLRs, NOD-like receptors; PAMPs, pathogen-associated molecular patterns; SCFAs, short-chain fatty acids; HDAC, histone deacetylase; GPCRs, G protein-coupled receptors; Tregs, regulatory T cells; MCFAs, medium-chain fatty acids; LCFAs, long-chain fatty acids; NHS, Nurses’ Health Study; PUFA, polyunsaturated fatty acid; MCT1, monocarboxylate transporter 1; SMCT1, sodium monocarboxylate transporter 1; DCs, dendritic cells; AhR, aryl hydrocarbon receptor; IELs, intraepithelial lymphocytes; GWAS, genome wide association studies; cAMP, cyclic-adenosine monophosphate; TJ, tight junction; ZO-1, zonula occludens-1; AMPs, antimicrobial peptides.
